# The effect of preference and actual days spent working from home on stress and musculoskeletal pain in older workers

**DOI:** 10.1007/s00420-023-01992-7

**Published:** 2023-07-19

**Authors:** Jodi Oakman, Katrina A. Lambert, Victoria P. Weale, Rwth Stuckey, Melissa Graham

**Affiliations:** 1 Centre for Ergonomics and Human Factors, School of Psychology and Public Health, La Trobe University, Bundoora, 3086 Australia; 2 School of Psychology and Public Health, La Trobe University, Bundoora, Australia 3086

**Keywords:** Ageing, Sense of community, Technology, COVID-19, Musculoskeletal pain, Stress

## Abstract

**Objectives:**

The rapid shift to working from home (WFH) due to the COVID-19 pandemic provided a unique opportunity to examine the relationship between preferred and actual days spent working from home on employees musculoskeletal pain (MSP) and stress in older workers.

**Methods:**

This study uses three waves of data from the Employees Working from Home (EWFH) study collected in May 2021 (*n* = 451), November 2021 (*n* = 358) and May 2022 (*n* = 320) during the COVID-19 pandemic. A generalised mixed-effect model was used to model the relationships between preference and actual days spent WFH, stress and MSP. Exploratory mediation analysis was conducted to further explore significant relationships between actual days WFH and outcomes.

**Results:**

WFH was associated with increasing stress levels in older participants, when the actual number of days WFH increased (B: 0.051, 95% CI: 0.008, 0.094) and when the number of days WFH exceeded their preferences (B: 0.218, 95% CI: 0.087, 0.349). Actual number of days spent WFH and stress in older employees was mediated through their sense of community (Indirect effect: 0.014, 95% CI: 0.003, 0.03; *p* = 0.006). The relationship between WFH and MSP was variable. For older employees, WFH more than their preferred number of days was associated with a higher likelihood of reporting MSP (OR: 4.070, 95% CI: 1.204, 13.757).

**Conclusions:**

Findings from this study support the need for flexible policies to support WFH which take into account employees preferences. For older workers, a sense of community was found to be important and proactive attempts to restore this will be important for maintain their health and supporting sustainable employment.

Teleworking or working from home (WFH) has existed since the 1970s (Eurofound and the International Labour Office 2017). However, the declaration of the COVID-19 pandemic in March 2020 (Ghebreyesus 2020), resulted in a dramatic rise in the number of employees WFH, as part of widespread mitigation strategies to reduce the spread of the virus (Kniffin et al. 2021). Although the overall number of people WFH had steadily increased prior to 2020, for many the rapid shift due to COVID-19 instigated a significant and unanticipated change to working arrangements, also referred to as a career shock (Akkermans et al. 2020). In Australia, like other countries, the COVID-19 pandemic led to a rapid shift to working from home, and for many, the blurring of boundaries between work and home life (Oakman et al. 2022a, b; Syrek et al. 2022). The restrictions varied across the country, one state (Victoria) had public health restrictions concerning WFH over an extended period of two and a half years. Mandated WFH was introduced in March 2020, before being replaced with a strong recommendation to WFH in May 2020, but very few workers returned to the office. The WFH mandate was reinstated in July 2020. In December 2020, a staged return of office workers was announced; however, the mandated WHF orders were quickly reinstated in February 2021. This WFH mandate was replaced with a recommendation to WFH in November 2021 (Victorian State Government 2021) and was finally lifted in September 2022, with organisations dealing with a return to the office in a variety of ways. Knowledge on the impacts of WFH on employees’ health and well-being is increasing as the world is adjusting to the significant changes to more traditional models of work where the shared office was considered the principal location. However, more nuanced analysis of different employee groups, such as older workers, is needed (Akkermans et al. 2020) to identify opportunities to optimise the WFH experience and reduce any negative health consequences that may arise.

An ageing population is, and will continue to have, a significant impact on the available labour force (Bloom et al. 2015). This situation is exacerbated by the COVID-19 pandemic, which has resulted in additional challenges to the worker supply in many countries, including Australia (Zarghami 2021). The Organisation for Economic Cooperation and Development (OECD) (2020) has proposed a need for longer working lives and stronger incentives to encourage workers to remain at work. The OECD report (2020) emphasises telework or WFH needs to be “carefully designed to meet the needs of workers and employers and maximise worker well-being and productivity (p. 11)”. However, longer working lives also requires workers to have sufficient fitness for work to sustain their careers. Previous research has shown older workers to be at risk of early retirement due to a range of health conditions (Leijten et al. 2015; Palmer and Goodson 2015) including MSP. As WFH and hybrid work becomes embedded into accepted work arrangements, this may offer opportunities for older workers to enable greater workplace accommodations and prolonged working lives. However, more information is needed to inform the development of organisational responses to older age-specific concerns associated with the COVID-19 pandemic and its impact (Kniffin et al. 2021).

The relationship between exposure to workplace physical and psychosocial hazards and employees’ stress levels and musculoskeletal pain (MSP) has been widely documented (Eatough et al. 2012; Hauke et al. 2011; Lang et al. 2012; Leka and Kortum 2008; Long et al. 2012; Niedhammer et al. 2021; Siegrist and Wege 2020). However, most research has been undertaken in situations where workers and their managers are co-located. Since the pandemic, a number of studies have explored WFH and different aspects of employee health (Bosma et al. 2022; Galanti et al. 2021) but specific analysis of older workers is limited. One systematic literature review and meta-analysis undertaken prior to the pandemic reported that older workers perceived WFH as less advantageous than office-based arrangements (Nakrošienė et al. 2019). The issue of preference on work location and its influence on employee health is a gap and of relevance to inform future design of work as suggested by the OECD (2020).

One challenge, identified in a systematic review on the health effects of WFH, was that of maintaining connection with colleagues (Oakman et al. 2020), particularly when relationships were typically formed through being co-located. This connection or sense of community refers to the relationships between workers and their colleague and their managers. Previous research has identified sense of community as related to employees’ general health (Graham et al. 2023), and emerging evidence suggests that for those WFH, maintaining a sense of community can be challenging, but to date this has not been examined in the context of employee preference for WFH and whether age differences exist in these relationships.

Technology has enabled the adoption of more flexible work arrangements; however, despite the benefits of being able to work in a range of settings, it has also been associated with negative impacts on employees’ mental health. The term technostress refers to adverse results from interactions between technology and employees and was proposed in the 1980s as a cost associated with the computer revolution (Brod 1984). Technostress can arise due to knowledge gaps between workers and the technology required for the job, or overload from increased expectations of being available to manage work beyond conventional working hours (Dragano and Lunau 2020). The COVID-19 pandemic disrupted normal patterns of work and required many to learn new skills to manage WFH and negotiate new ways of working, which for many required a negotiation of how they incorporated work into their home environment and a resetting of boundaries. The concept of person–environment (PE) fit, a well-established framework which proposes attitudes, behaviours and other individual-level outcomes result not from just the person or their environment but rather from the relationship between the two (Caplan 1987). PE fit is proposed to offer a way to conceptualise the role of preferences on employees’ stress levels and MSP, as a match between preference and actual days WFH will result in improved fit and may reduce any negative impacts on workers’ health. Several gaps have been outlined in the current knowledge on the impact of WFH on older workers and their physical and mental health. Our hypothesis for the current study is that a mismatch in employee preference and actual days WFH may result in a stress response with subsequent impacts on older workers’ mental and physical health.

This study will specifically examine the following research question:

Are the relationships between actual and preferred days of WFH and employees’ stress or MSP the same in older workers compared to younger workers?

## Methods

### Setting and design

This study uses three waves of data from the Employees Working from Home (EWFH) study (Oakman et al. 2022a, b) collected in May 2021 (*n* = 451, 67% response rate), November 2021 (*n* = 358, 53.4%) and May 2022 (*n* = 320, 47.8%). The baseline data of the EWFH study (collected in October 2020 during strict COVID-19 lockdown) were not utilised as information regarding WFH preference was not asked and a mandated WFH order applied to the majority of the sample. All respondents were required to be working from home at least 2 days a week.

### Measures

#### Exposures

Actual number of days worked from home was asked by the survey item “How many days of the week are you currently working at home?” with six options ranging from 0 to “5 or more”. Working from home preference was asked by the item “Taking everything into account, how many days per week would you prefer to work at home?” with six options ranging from 0 to “everyday”. Variation between actual and preferred WFH arrangements were calculated in two ways: (1) absolute difference—a positive integer representing the difference between the number of days preferred to be WFH and the number of days actually WFH and (2) whether the respondent is WFH more, less, or their preferred number of days. Organisational support for WFH arrangements was measured by the question “How supportive is your organisation in allowing you to choose your location of work (e.g. working at home, some days at home with some in the office)?” with five options ranging from “to a very small extent” to “to a very large extent”.

#### Outcomes

Stress was measured using 13 items from the Copenhagen Psychosocial Questionnaire (COPSOQ) scored on a five-point scale ranging from not at all (1) to all the time (5) (Burr et al. 2019).

Musculoskeletal discomfort/pain frequency and severity ratings were recorded separately for five body regions (neck/shoulders, hands/fingers, arms, middle to lower back, and hips/bottom/legs and feet) using a measure with evidence of validity from a range of different industry sectors (Oakman et al. 2014). Response options for pain/discomfort frequency ranged from never (0) to almost always (4). Severity, if applicable, was scored using a three-point scale from mild (1) to severe (3). Respondents were considered to have pain presence if they reported any pain. For those with pain, a pain score was derived by multiplication of frequency by severity for each body region and adding the resulting scores, creating a scale from 1 to 60, using a previously described and published method (Oakman and Chan 2015).

#### Potential mediators

Workplace sense of community and social support were measured using items from the Copenhagen Psychosocial Questionnaire (COPSOQ; Burr et al. 2019) with items rated on a five-point scale from never/hardly ever (1) to always (5). Both constructs were measured by two items and had good reliability—an example item for sense of community (Spearman–Brown = 0.82) is “Is there a good atmosphere between you and your colleagues?"; an example item for social support (Spearman–Brown = 0.89) is “My colleagues are willing to listen to my problems if needed”. The full survey is reported elsewhere (Oakman et al. 2022a, b).

Technological support was measured by three items (example item—“I can get good help and support from work if I have technology (hardware or software) problems”; Cronbach α = 0.72) rated on a five-point scale from strongly disagree (1) to strongly agree (5). Issues with technology were measured by agreement to two statements, “The technical hardware I use when working at home (e.g. laptop phone) enables me to work effectively” and “The software I use when working at home enables me to work effectively” rated on a five-point scale from strongly disagree (1) to strongly agree (5). These statements were examined as standalone items and then combined (Spearman–Brown = 0.87).

#### Covariates

Age was based on the question “What is your age group?” 18–25 years; 26–35 years; 36–45 years; 46–55 years; 56 years and over. Age groups were collapsed into younger (45 and under) and older workers (46 and over). Gender was based on the question “Are you: Male, Female, Other”, the five (0.9%) persons who identified as Other were excluded from this analysis. Participants were classified as having children at home during work hours if they answered “yes” to the question “When you are working at home are children usually at home with you?” in any survey. Home workspace location was based on the item “When you are working at home, where do you usually work?” Three response options were provided and coded as follows: Wherever—“I just find a place somewhere that's free, such as on the kitchen table or other place”; Separate—“I have my own place in a separate room by myself”; and Interruptions—“I have my own place but in a room that can be busy with other people.”

### Statistical analysis

The three waves of data collection were compared in terms of demographic data, and actual and preferred WFH patterns. A generalised mixed-effect model with gaussian link function and random slope ID was used to model the relationships between preference and actual days spent WFH and stress. The presence of MSP was similarly modelled using generalised mixed-effect model, binomial link and random ID. Odds Ratios (OR) were calculated to facilitate interpretation of results. The overall pain scores (1–60) were modelled with a log link and negative binomial distribution to allow for the estimation of under dispersion or overdispersion. Estimation of dispersion avoids reliance on an assumption that the mean and variance of the outcome are equal. Rate Ratios (RR) were calculated to facilitate interpretation. The RR represents the change in the pain score in terms of percentage per unit increase of continuous independent variables. The analysis was stratified by dichotomised age of participant (≤ 45 or 46 +). Exploratory mediation analysis was conducted to further explore significant relationships between actual days WFH and outcomes. Calculation of direct and indirect effects and proportion of mediation was completed using the R package “mediation” (Tingley et al. 2014).

All analysis was conducted in R version 4.1.3 “One Push-Up” (R Core Team 2021).

## Results

Table 1 shows characteristics of participants. Over 75% of the respondents were women. Although many reported having children (31.4–34.6%), these were identified as not usually present while respondents were WFH. As expected, the actual number of days WFH varied substantially over the survey time period, with the median number of days WFH during wave 2 reported as 5. However, overall preference in the number of days WFH did not substantively change over the period of analysis.

**Table 1:** Demographics of participants and preferred WFH patterns across 3 waves of data

	Wave 1 (*n* = 451)	Wave 2 (*n* = 358)	Wave 3 (*n* = 320)
Gender			
Male	103 (22.8%)	84 (23.5%)	65 (20.3%)
Female	348 (77.2%)	274 (76.5%)	255 (79.7%)
Age			
18–35 years	115 (25.5%)	82 (22.9%)	63 (19.7%)
36–45 years	134 (29.7%)	102 (28.5%)	89 (27.8%)
46–55 years	128 (28.4%)	110 (30.7%)	101 (31.6%)
56 + years	74 (16.4%)	64 (17.9%)	67 (20.9%)
Children			
No	295 (65.4%)	245 (68.4%)	212 (66.3%)
Yes	156 (34.6%)	113 (31.6%)	108 (33.8%)
Child present			
No	433 (96.0%)	288 (80.4%)	305 (95.3%)
Yes	18 (4.0%)	70 (19.6%)	15 (4.7%)
Actual days WFH			
Mean ± SD	2.441 ± 1.697	4.014 ± 1.520	2.428 ± 1.596
Median [IQR]	3 [2]	5 [2]	3 [2]
Preferred no of days WFH			
Mean ± SD	2.714 ± 1.340	2.726 ± 1.336	2.756 ± 1.366
Median [IQR]	3 [2]	3 [2]	3 [2]
Actual verses preferred days WFH			
Equal	159 (35.3%)	66 (18.4%)	134 (41.9%)
WFH more than wish to	101 (22.4%)	250 (69.8%)	60 (18.8%)
WFH less than wish to	191 (42.4%)	42 (11.7%)	126 (39.4%)
Variation in preference (days)			
0	159 (35.3%)	66 (18.4%)	134 (41.9%)
1	174 (38.6%)	98 (27.4%)	116 (36.3%)
2	76 (16.9%)	114 (31.8%)	54 (16.9%)
3	35 (7.8%)	63 (17.6%)	13 (4.1%)
4	3 (0.7%)	11 (3.1%)	1 (0.3%)
5	4 (0.9%)	6 (1.7%)	2 (0.6%)
Organisation supports WFH			
To a very small extent	45 (10.0%)	28 (8.2%)	17 (5.4%)
To a small extent	37 (8.2%)	23 (6.7%)	25 (7.9%)
Somewhat	101 (22.4%)	95 (27.9%)	86 (27.1%)
To a large extent	156 (34.6%)	98 (28.7%)	103 (32.5%)
To a very large extent	112 (24.8%)	97 (28.4%)	86 (27.1%)

As the overall variation increased between actual number of days WFH and preferences for WFH, a modest increase in stress levels for all participants was found (B: 0.062, 95% CI: 0.028, 0.097) (Table 2). WFH was associated with increasing stress in older participants, when the actual number of days WFH increased (B: 0.051, 95% CI: 0.008, 0.094) and when the number of days WFH exceeded their preferences (B: 0.218, 95% CI: 0.087, 0.349). Employees who reported their organisation as supporting WFH arrangements were more likely to have lower stress levels, but when stratified by age this association was attenuated.

**Table 2:** Associations between preference verses actual number of days WFH and stress

	Total sample (1129 obs) B (95% CI)	Age ≤ 45 (585 obs) B (95% CI)	Age 46 + (544 obs) B (95% CI)
Actual days WFH	0.029 (− 0.001, 0.059)	− 0.001 (− 0.042, 0.040)	0.051 (0.008, 0.094)
Preferred days WFH	− 0.028 (− 0.066, 0.008)	− 0.056 (− 0.108, − 0.003)	0.002 (− 0.050, 0.054)
Actual verses preferred days WFH			
Match	Reference	Reference	Reference
WFH > prefer	0.143 (0.055, 0.232)	0.093 (− 0.027, 0.214)	0.218 (0.087, 0.349)
WFH < prefer	− 0.007 (− 0.099, 0.085)	0.031 (− 0.102, 0.164)	− 0.012 (− 0.140, 0.115)
Absolute variation	0.062 (0.028, 0.097)	0.065 (0.017, 0.113)	0.055 (0.005, 0.105)
Organisation supports WFH	− 0.031 (− 0.069, − 0.006)	− 0.025 (− 0.077, 0.027)	− 0.048 (− 0.103, 0.001)

All models adjusted for gender, children present during work hours, home workspace location and survey timing. A random effect for each participant is included

**Fig. 1:**
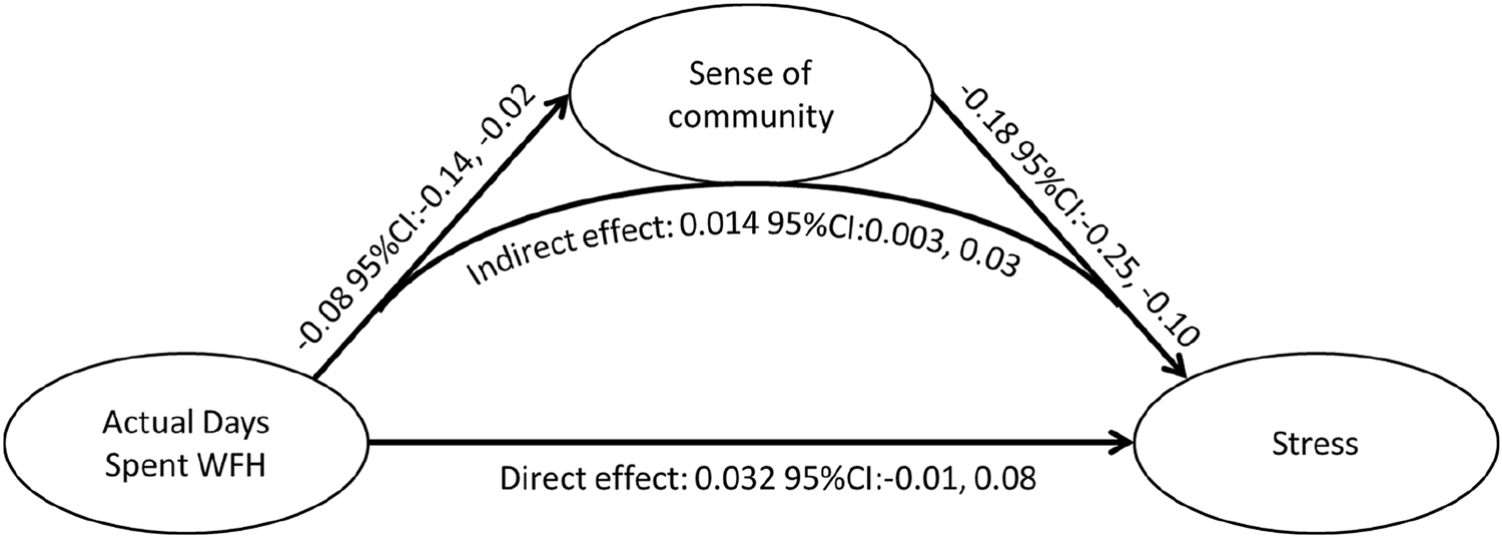
Mediation model of effects of the association between actual days spent working from home and stress in older adults

Analysis of significant relationships between actual days WFH and stress found that 28% of the association between actual number of days spent WFH and stress in older employees was mediated through their workplace sense of community (Indirect effect: 0.014 95% CI: 0.003, 0.03; *p* = 0.006). Increasing the actual number of days WFH in older adults was associated with a decrease in their workplace sense of community, which in turn was associated with an increase in stress levels (Fig. 1). The same relationship was not observed for younger workers. The relationship between WFH and stress in older adults was not significantly mediated by technological support, issues with technology or social support from colleagues (data not shown).

The presence of MSP was not consistently associated with WFH (Table 3). For older employees, WFH more than their preferred number of days was associated with a higher likelihood of reporting MSP (OR: 4.070 95% CI: 1.204, 13.757). Increasing the number of preferred days WFH was associated with a reduction in the likelihood of younger employees reporting MSP (OR: 0.692, 95% CI: 0.481, 0.995). No associations between WFH preference and actual days spent working from home were identified with the severity of musculoskeletal pain (Table 4).

**Table 3:** Associations between preference verses actual number of days WFH and MSP

	MSP (total sample) (1129 obs) OR (95%CI)	Age ≤ 45 (585 obs) OR (95%CI)	Age 46 + (544 obs) OR (95%CI)
Actual days WFH	0.950 (0.672, 1.343)	1.285 (0.842, 1.960)	0.612 (0.334, 1.121)
Preferred days WFH	0.830 (0.636, 1.084)	0.692 (0.481, 0.995)	1.050 (0.657, 1.678)
Actual verses preferred days WFH			
Match	Reference	Reference	Reference
WFH > prefer	1.900 (0.923, 3.909)	1.152 (0.448, 2.962)	4.070 (1.204, 13.757)
WFH < prefer	1.211 (0.596 2.464)	0.750 (0.283, 1.988)	1.962 (0.637, 6.047)
Absolute variation	1.147 (0.869, 1.514)	0.903 (0.621, 1.312)	1.745 (1.063, 2.863)
Organisation supports WFH	0.959 (0.720, 1.279)	1.039 (0.709, 1.522)	0.833 (0.511, 1.357)

All models adjusted for gender, children present during work hours, home workspace location and survey timing. A random effect for each participant is included

**Table 4:** Associations between preference verses actual number of days WFH and level of MSP

	MSP LEVEL (total sample) (802 obs) RR (95%CI)	Age ≤ 45 (426 obs) RR (95%CI)	Age 46 + (376 obs) RR (95%CI)
Actual days WFH	1.012 (0.984, 1.040)	1.029 (0.993, 1.065)	0.993 (0.950, 1.037)
Preferred days WFH	0.983 (0.950, 1.017)	0.999 (0.953, 1.047)	0.961 (0.915, 1.009)
Actual verses preferred days WFH			
Match	Reference	Reference	Reference
WFH > prefer	0.995 (0.917, 1.080)	0.986 (0.882, 1.103)	1.057 (0.932, 1.199)
WFH < prefer	0.942 (0.864, 1.027)	0.933 (0.820, 1.061)	0.962 (0.853, 1.086)
Absolute variation	1.006 (0.974, 1.038)	1.013 (0.969, 1.059)	1.002 (0.956, 1.050)
Organisation supports WFH	1.002 (0.966, 1.038)	0.983 (0.936, 1.033)	1.025 (0.971, 1.083)

All models adjusted for gender, children present during work hours, home workspace location and survey timing. A random effect for each participant is included

## Discussion

The overall aim of the current study was to examine whether the relationships between actual and preferred days of WFH and employees’ stress or MSP are same in older workers compared to younger workers? The relationship between WFH and stress was further explored to identify other influences, such as workplace sense of community, and the use of technology on the relationship. When the number of days spent WFH increased and where a mismatch between preferred number of days and the actual number of days spent WFH existed, older workers reported higher stress levels than their younger colleagues. The relationship between the number of days worked from home and stress was mediated by workplace sense of community for older workers. In relation to WFH and MSP, older workers who were WFH more than their preferred number of days were more likely to report MSP. The severity of the MSP was not influenced by either the number of days spent working for home or a mismatch in preference for older workers.

The differences in preferences for WFH days observed in the current study may offer insights into future human resource practices and how to optimise work to reduce negative impacts of older employees’ health. Clear differences were identified in the preferences for the number of days spent WFH, and the subsequent impact of a mismatch in that preference on older workers’ stress levels and MSP. Previous research has identified a relationship between WFH and increase reporting of MSP during the COVID-19 pandemic (Bosma et al. 2022; Oakman et al. 2022b) but did not specifically examine age-related differences. In relation to stress, the results are mixed potentially due to the significant contextual differences between countries, industry settings and management styles but as with MSP age-related differences were not specifically examined (Chirico et al. 2021; Oakman et al. 2023).

Traditional models of work have relied on “line of site” management styles where managers and supervisors are co-located and can identify issues, actual or potential, in real time (Kniffin et al. 2021). Clearly, this is not possible in remote working and has required adjustment from managers and supervisors. The differences in reported stress levels for older workers, who may have been working in more traditional models for more years than their younger colleagues, may reflect that the imposed adjustment to WFH may be more challenging, particularly as it was initially implemented without employee input.

Maximising PE fit (Caplan 1987) has long been proposed as a strategy to promote sustainable employment and extended working lives (Oakman and Wells 2016). Flexible work practices are one strategy through which employees can have choice over their working hours and the location of work, which includes WFH, offers choices to support an individual to work optimally (Skinner et al. 2014). However, the context of previous research has been undertaken in a pre-COVID-19 pandemic environment where WFH was typically more constrained and limited to one or two days a week usually through negotiation and sometimes for a defined period (Gajendran and Harrison 2007). The pandemic has changed perceptions of the possibilities for WFH and the current study suggests that accommodating employee preferences, particularly for older workers, is likely to result in reduced stress levels and MSP for those workers. Kooij (2020) proposed that older adults are engaged in self-regulation strategies aimed at continuously maintaining PE fit to enable successful ageing at work and the dynamic nature of PE fit is also supported by others (Kim et al. 2020). Our findings would suggest that organisations would benefit from supporting older workers in utilising these strategies through appropriate policy settings which afford some autonomy over work location and workers’ ability to choose when and where they work. Older workers are a heterogenous group, a factor which can be accommodated through the use of a PE fit framework which supports enabling workers to be engaged in the design of their job or job crafting, including physical and psychosocial working conditions, which can support improved PE fit (Kooij et al. 2017) and supports previous research identifying that workers needs are different at various life stages (Skinner et al. 2014).

In relation to the actual numbers of days worked from home, workplace sense of community was an important factor in reports of higher stress levels for older workers, suggesting that they may value being co-located with their colleagues for interaction and support. As a result, organisations will need to consider strategies to facilitate connections as with increased use of hybrid work models, as opportunities for in-person connections with colleagues at work will be more limited than prior to the start of the pandemic. As the impacts of the pandemic continue and we need to maintain flexibility with models of work, it is important that we draw on evidence from workers and consider how to ensure optimising PE fit is enabled through workplace policies and practices, and in line with governmental strategies which are specifically acknowledging flexibility of work (Safe Work Australia 2023). As we enter the fourth year of the pandemic, ensuring sustainable employment conditions which promote health of older workers is important to contribute towards addressing the ongoing labour supply issues that appears to be part of our new normal. Further longitudinal evaluation of hybrid working patterns and examining the impact of WFH workplace policies and practices on employees mental and physical health is needed.

### Strengths and limitations

The longitudinal design of this study is a key strength, with three waves of data collected over a 12-month time period during the COVID-19 pandemic. The study design enabled investigation of how older workers’ preferences for number of days WFH influenced their stress levels and MSP. In addition, it enabled analysis of the role of workplace sense of community in the reporting of older workers’ stress levels. However, some limitations arise as with all studies, the first being that data related to workplace sense of community, stress levels and MSP were not collected prior to the COVID-19 pandemic. In addition, the strategies employed by organisations prior to the pandemic are not known. The higher proportion of females compared with males in the sample is consistent with other COVID-19 research, and study retention rates may limit the generalisability of the findings. As such, the results should be interpreted with caution. Population-level data on those working from home are not currently available. To our knowledge, this study is the first to examine aged related differences between WFH, stress and MSP.

## Conclusion

The current study used longitudinal data collected during the COVID-19 pandemic providing an opportunity to identify differences in older workers’ WFH preferences and actual days worked at home on their stress and MSP levels. The findings provide insights into the needs of older workers and the requirements by organisations to support WFH as we continue to operate in hybrid models of work. To optimise older workers stress levels and MSP, some flexibility will be required to navigate the tension between their preference for days WFH and the organisational requirements. Findings from this study support the need for organisations to focus on ensuring opportunities exist for collaboration and contact with colleagues whilst WFH to reduce the potential for increased stress levels. Further, older workers prefer more office days than their younger colleagues which suggests nuance is required in workplaces policies and procedures to accommodate individual differences in working locations to ensure optimisation of employees’ health.

## Data Availability

Data will be made available on reasonable request.
